# Characterization of four *Acidovorax* phages and their potential in phage biocontrol for lamb’s lettuce seed decontamination

**DOI:** 10.1128/spectrum.00993-24

**Published:** 2024-10-28

**Authors:** Dominique Holtappels, Francisca G. Vieira, Marleen Voet, Marta Vallino, Johan Van Vaerenbergh, Rob Lavigne, Jeroen Wagemans

**Affiliations:** 1 Laboratory of Gene Technology, Biosystems, Leuven, KU Leuven, Belgium; 2 Institute for Sustainable Plant Protection, National Research Council of Italy, Turin, Italy; 3 Flanders Institute for Agriculture and Fisheries, Ghent, Belgium; The University of Tennessee Knoxville, Knoxville, Tennessee, USA

**Keywords:** phage biocontrol, *Acidovorax*, seed bioassay

## Abstract

Bacterial black spot, caused by *Acidovorax valerianellae*, is responsible for significant yield losses in lamb’s lettuce (*Valerianella locusta*) in many producing countries, especially Europe. Currently, no resistant varieties of *V. locusta* are available that effectively control the disease under field conditions. Bacteriophage-based biocontrol has been suggested as a sustainable and natural alternative strategy to combat bacterial pathogens. In this study, novel phages infecting *A. valerianellae* and *Acidovorax cattleyae*, Alfacinha1, Alfacinha3, Acica, and Aval, were isolated and characterized. Based on comparative genomics, these phages represent three new phage genera. Aval and Acica phages revealed genomic features characteristic of temperate lifestyle, encoding toxins likely associated with lysogenic conversion, which contrasts with Alfacinha1 and Alfacinha3. The latter was selected for application as a biocontrol agent during seed steeping. This phage reaches an 87% reduction in the *A. valerianellae* concentration on artificially infested seeds. Importantly, this reduction results in an increased germination rate from 58.9% to 93.3%. Moreover, the infected seedlings had a dramatic reduction in vigor index after 22 days of growth, whereas the phage-treated ones had a vigor index similar to the negative control, reinforcing the ability of bacteriophages to effectively reduce disease progression. We further evaluated the impact of lipopolysaccharides in phage suspensions on the development of seedlings. Here, we demonstrated that the presence of lipopolysaccharides do have an impact on seedling development, significantly reducing the number of roots developed. This study shows how genomic analyses and tailored bioassays represent an essential route to ensure safe phage application and demonstrates the potential of a phage-based biocontrol strategy against *A. valerianellae*.

**IMPORTANCE:**

Bacteria continue to globally cause serious damage to a variety of crops. One example is a bacterial black spot of lamb’s lettuce caused by *Acidovorax valerianellae*. It has spread across Europe, resulting in economic losses of at least 10% in tonnage annually. Faced with the inefficiency of conventional control methods, an alternative and sustainable strategy based on the use of bacteriophages was pursued in this study. We present for the first time the isolation and characterization of *A. valerianellae*-specific phages. Moreover, we assessed their biocontrol potential in seed decontamination since the disease primarily spreads from seeds to seedlings. Interestingly, seed treatment with one of our phages reaches an 87% reduction in bacterial concentration. More importantly, this reduction results in an increased germination rate from 58.9% to 93.3%. Finally, our study demonstrated for the first time the need for removing endotoxins from phage suspensions as they impact plant development when used as a biocontrol agent.

## INTRODUCTION

The *Acidovorax* genus was established in 1990 and initially contained only non-phytopathogenic species *Acidovorax facilis*, *Acidovorax temperans*, and *Acidovorax delafeldii* (1). The plant pathogenic members of the genus, originally classified within the *Pseudomonas* genus, were transferred to the *Acidovorax* genus in 1992 by Willems et al. (2). The genus belongs to the beta division of the Proteobacteria (1) and includes a variety of species that exhibit distinctive lifestyles. From the species described in this genus, it is shown that some are well adapted to water and soil environments whereas others interact with eukaryotic organisms, acting primarily as phytopathogens. Among the latter, species were found to cause disease to a variety of agriculturally and economically important crops including *Acidovorax citrulli*, *Acidovorax avenae*, *Acidovorax oryzae*, *Acidovorax cattleyae*, *Acidovorax konjaci*, *Acidovorax anthurii*, and *Acidovorax valerianellae* (2–5).

*A. valerianellae* causes bacterial black spot of lamb’s lettuce (*Valerianella locusta*), also called corn salad (6). In Asia, it was reported that *A. valerianellae* can also infect watermelon (7, 8), tea (9), and hydrangea (10). The disease was first observed in fields in the West of France in 1991, and since then, it has spread to several other countries across Europe, resulting in economic losses in corn salad cropping of at least 10% in tonnage annually (11, 12). Typical disease symptoms appear as black spots on cotyledons, leaves, petioles, and stems. Cotyledons and leaves initially develop water-soaked, angular lesions, which later develop into black necrotic spots. In the case of severe infection, foliar lesions may also coalesce into blights (13). In recent years, fresh-cut vegetables have attracted consumer interest as a practical alternative to traditional vegetable crops presenting a wide number of advantages, such as freshness, safety, convenience, and labeled information (14). These factors often result in increased prices and so to economic benefits for those involved in the production chain (15). Consequently, the production of ready-to-eat leafy vegetables has gained increasing importance in Europe. However, Black spot disease reduces corn salad quality significantly, especially in the fresh market, making the affected batches unmarketable (12).

The period between infection and symptom expression ranges from 3 to 21 days and is influenced by temperature, plant age, and soil characteristics, including the nutrient status of the plants. *A. valerianellae* colonizes leaves and roots shortly after seed germination and can be detected even without symptom development (13). Transmission by contaminated seeds and soil is discussed as a major infection sources (16). Bacteria released from leaf and stem lesions in exudates can also be spread via splashing water and wind-driven rain, facilitating short-distance pathogen dispersal, entering the host tissue through natural openings, such as stomata, or wounds possibly caused by agronomic operations (13). During the late stages of lamb’s lettuce production, when plants are densely planted and overhead watering is used, this poses a major risk for the development of a black spot pandemic. Between lamb’s lettuce plantings, *A. valerianellae* persists in contaminated seeds and infected plant debris in the soil and can be recovered from soil for up to 39 days after harvest of a diseased crop (11). Continuous cultivation raises soil inoculum levels, which lead to infection of subsequent crops during germination and other plant growth stages.

As *Acidovorax* pathogens have shown resistance to antibiotics (17), and because its overuse has been raising public concern, alternative control strategies are urgently needed. A wide range of seed treatments, including thermotherapy, chemical, and biochemical methods, have been conducted for the control of *Acidovorax* pathogens, such as *A. citrulli* and *A. valerianellae*. However, it remains a big challenge to fully decontaminate the infested seeds, once the pathogen is located in the embryo, where is protected from externally added antimicrobial compounds (18, 19). For instance, fermentation of cucurbitaceous seeds with chitosan, streptomycin sulfate, sodium hypochlorite, peroxyacetic acid, mercury chloride, hydrochloric acid, and calcium chloride has been reported to significantly reduce seed-to-seedling transmission of *A. citrulli* (18, 19). In lamb’s lettuce specifically, several seed disinfection methods were tested, including aerated steam, hot water, sodium hypochlorite, ethanol, and calcium hydroxide (20). Although sodium hypochlorite was revealed to have an effect against *A. valerianellae*, it is not allowed in organic farming since it presents poor degradability and potential toxicity (21).

Rahimi-Midani and Choi demonstrated the potential of phage biocontrol to control bacterial fruit blotch (BFB) of cucurbit crops. Novel bacteriophages for *A. citrulli*, named as ACP17 (22) and ACPWH (23), were fully characterized and tested by both seed coating (23) and soil-based (24) plant assays. Coating of watermelon seeds with the bacteriophages ACP17 and ACPWH enhanced plant germination and survival, modulating the progression of BFB (24). To date, however, no phages were reported that infect *A. valerianellae* specifically.

In this research, we present the isolation and characterization of four novel *Acidovorax* phages. We taxonomically categorized the phages using comparative genomics and determined the lifestyle of the phages by means of microbiological assays. The biocontrol potential of the most promising phage, active against *A. valerianellae*, was evaluated in a seed bioassay. Finally, the impact of the presence of lipopolysaccharides in phage suspensions on the development of seedlings was evaluated.

## MATERIALS AND METHODS

### Phage isolation

Phages were isolated from soil samples collected from *Acidovorax*-infected greenhouses in Flanders, Belgium. Bacterial strains were grown overnight in lysogeny broth with reduced salt concentration (1.5 g/L, 1 mL) distributed in deepwell plates at 25°C while shaking. Soil samples were added (1 g) and incubated overnight. Afterward, a drop of chloroform was added to the well and incubated for 1 hour before centrifugation and filtration of the enriched sample. The filtrate was spotted (3 µL) on a bacterial lawn corresponding to the host to which the sample was enriched. Lysis zones were picked up with sterile toothpicks and suspended in 100 µL of phage buffer (10 mM Trizma base, 10 mM MgSO_4_, and 150 mM NaCl; pH 7.5). These samples were plated using the double agar technique. Plaques originating from these samples were picked up and plated. To obtain pure phage stocks, this process was repeated three consecutive times. Throughout the rest of the assays, bacteria were grown in/on Pseudomonas *P* broth/agar (20 g/L bacto peptone, 1.4 g/L MgCl_2_.6H_2_O, 10 g/L K_2_SO_4_, supplemented with 7 g/L, or 15 g/L Bacto agar for soft or solid agar).

### Phage characterization

Transmission electron microscope (TEM) pictures were captured as described by Martino and colleagues (25). The phage suspensions were adsorbed for 3 minutes on carbon and formvar-coated copper-palladium grids, which were then rinsed several times with water. The grids were negatively stained with aqueous 0.5% uranyl acetate, and the excess fluid was removed with filter paper. Observations and photographs were made with a Philips CM10 TEM (Eindhoven, The Netherlands) at 80 kV. Micrograph films were developed and digitally acquired at high resolution with a D800 Nikon camera. Finally, the images were trimmed and adjusted for brightness and contrast using the Fiji software (26).

Phage host range was determined by spotting 3 µL of phage lysate (10^3^, 10^4^, and 10^5^ plaque forming units (PFU)/mL) in a dilution series on a lawn of a putative host. Strains were considered susceptible in case individual plaques were observed throughout the dilution series.

Killing curves were established for the strain GBBC 3161 infected with Alfacinha3 at a multiplicity of infections (MOIs) of 0.1, 1, and 10. The bacterial culture was initially infected at OD_600_ of 0.3 and monitored every 10 min for 2 h and compared to an uninfected culture. The killing curves represent the average of three independent biological repeats.

The adsorption of Alfacinha3 on bacterial host GBBC 3161 was evaluated by infecting the host (OD_600_ of 0.3) with an MOI of 0.01. Immediately after infection, a 200 µL sample was taken and transferred into a Zymo-Spin IC column (Zymo Research) in a pre-cooled eppendorf tube and centrifuged for a few seconds. The filtered suspension, kept on ice, was titrated to determine the number of non-adsorbed phages. This was repeated after 1, 5, and 10 minutes after infection. Data represent three independent biological repeats.

### Whole-genome sequencing of the phage genomes

Phage DNA was extracted from a high-titer lysate (minimum of 10^8^ PFU/mL). One unit of DNase I and 1 U of RNase A were added to 10 µL of the phage stock. After incubation at 37°C for 30 minutes, 4 µL of EDTA (1.5 M), 5 µL of SDS 10%, and 1 µL of Proteinase K were added and subsequently incubated at 56°C in a thermal bath for 45 minutes. The Kit DNA Clean & Concentrator-5 (Zymo Research) was used to purify the phage DNA following the manufacturer’s instructions.

The genomic DNA was sequenced using Illumina MiniSeq platform at the Laboratory of Gene Technology, KU Leuven. A library was prepared using the Nextera Flex DNA Library Kit for each sample, according to the manufacturer’s guidelines. The quality of each library preparation was controlled using an Agilent Bioanalyzer 2100. All library preps were equally pooled and sequenced using a MiniSeq Mid Output flowcell (300 cycles; 2 × 150 bp reads). The reads were trimmed with the Trimmomatic tool (v0.36.5), using standard settings with the addition of an initial ILLUMINA CLIP step to remove the Nextera adapters (27). Next, the quality of the reads was assessed using the FastQC tool (v0.11.8) (28).

The raw read data gathered in 3.6.1 were processed (assembly and annotation) using online tools on the public servers of Galaxy (v21.05) (29) and PATRIC (v3.6.9) (30). The reads were assembled *de novo* using Unicycler (v0.4.8) (31). The assembled contigs were visualized, and their quality was assessed using Bandage (v0.8.1) (32). The automated annotation was manually curated by verifying the translated open reading frames (ORFs) in a BLASTp analysis (National Center for Biotechnology Information) (33) against the non-redundant GenBank protein database (34), and Artemis (v18.1.0) (35) was used to polish the genbank files. To classify the phages based on genome-wide similarities, a viral proteomic tree was generated with the online ViPTree server (v1.9) (36), and the intergenomic distances/similarities amongst the related viral genomes were computed using VIRIDIC web tool (37). Seaborn Python library (v0.11.1) (38) was used for the construction of the heatmaps. Easyfig (v2.2.2) (39) was used to create linear comparison figures of multiple genomes and BLAST comparisons between multiple genomic regions. MegaX (v10.2.4) (40) was used to conduct automatic sequence alignments, using the MUSCLE algorithm (41), and to construct phylogenetic trees according to the neighbor-joining method with 1,000 bootstraps.

### Seed bioassay *in vitro*

*V. locusta* seeds, “Groene van Cambrai” (Aveve, Belgium; 3 g), were first sterilized by suspending and shaking them for 7 minutes in 40 mL of a 1% NaClO solution using the HulaMixer Sample Mixer (Thermo Scientific) at 25 rpm and room temperature. The liquid was removed with a sterile pipette tip. Next, the seeds were rinsed three times with sterile mQ water (for 2 minutes) and left to dry under a laminar flow. The surface sterility of the seeds was tested on lysogeny broth (LB) agar plates (incubated at 30°C for 2 days).

The seeds were infected with GBBC 3161 at OD_600_ = 0.15 (around 10^8^ CFU/mL) and incubated for 1.5 hours, while shaking using the HulaMixer at 25 rpm and room temperature. Following seed drying, Alfacinha3 phage solution was added to the seeds with a concentration of 10^9^ PFU/mL and shaken overnight using the HulaMixer at 25 rpm at 16°C. Seeds were crushed and suspended in a phage buffer. The bacterial concentrations were quantified by plating on standard LB agar plates. The quantification of phages was performed using the soft agar overlay method. Overall, three independent repeats were performed for each one of the four different conditions (negative control, phage only, bacteria only, and bacteria plus phage).

### Seedling bioassays

Similarly as described above, lamb’s lettuce seeds were surface sterilized, inoculated with *A. valerianellae* GBBC 3161 (OD600 = 0.15) for 1.5 hours and phage primed (Alfacinha3) overnight. After priming, seeds were dried under a laminar flow. Using sterilized tweezers, 30 seeds per condition were sown onto plant growth medium, 1/4 Murashige and Skoog (MS) agar (1.1 g/L MS basal medium; 15 g/L bacteriological agar), in each plate. The plant growth medium enclosed half of the plates so that the seedling started growing parallel to the plate bottom. Furthermore, plates were sealed with Parafilm (Sigma-Aldrich) to contain the moisture and were placed vertically under a lamp providing light for 16 hours and 8 hours of darkness per day. For each condition, three independent repeats were performed, totaling to 90 seeds for each condition that were sown. After 22 days, the shoot and root length of each individual seedling were measured using a caliper.

In a second seedling bioassay, the effect of the presence of LPS in the phage preparation on seedling development was evaluated in a similar way. For this experiment, Alfacinha1 was used as phage, while *A. valerianellae* GBBC 3357 was used as inoculum. Six conditions were evaluated with 12 seeds per object: negative control—no bacteria or phage; bacteria only control; unpurified (no EndoTrap) phage only control; purified (with EndoTrap) phage only control; bacteria and unpurified phage sample; and finally, the bacteria and purified phage sample. For inoculation, the seeds were again primed for 1.5 hours in a bacterial solution (sterile Pseudomonas *P* broth was used for the controls), while the phage treatment was now done for 2 hours (sterile phage buffer was used for the controls). A volume of 3.5 mL was applied per 12-seeds batch. After sowing, the seedlings were allowed to grow on MS agar for 4 weeks. Again, three independent repeats were evaluated.

For amplification and purification of Alfacinha1 for this last bioassay, a 250 mL *A*. *valerianellae* GBBC 3357 culture (OD_600_ of 0.3) was inoculated with phage at an MOI of 0.01 and incubated overnight at 25°C. The culture was centrifuged at 4,000 *g* for 45 minutes at 4°C, and the supernatant was filtered with a 0.45 polyethersulfone (PES) filter. Next, the phages were concentrated by high-speed centrifugation at 35,000 *g* for 90 minutes and resuspension in 1:10 of the initial volume by overnight shaking at 4°C. The next day, the phages were gently pipetted up and down to further resuspend them. One batch was further purified to remove LPS endotoxins using an EndoTrap HD purification column (Lionex) following the manufacturer’s instructions, while another batch was kept unpurified (no EndoTrap purification).

### Statistical analysis

Statistical analyses were performed with JMP Pro 15. Multiple non-parametric Wilcoxon comparison tests and Tuckey-Kramer HSD (honestly significant difference) for multiple comparisons were performed at a significance level of 0.05. For statistical data visualization, such as the design of the box plots, Seaborn (v0.11.1) was used (38).

## RESULTS

### Isolation of bacteriophages and their host range

**Fig 1:**
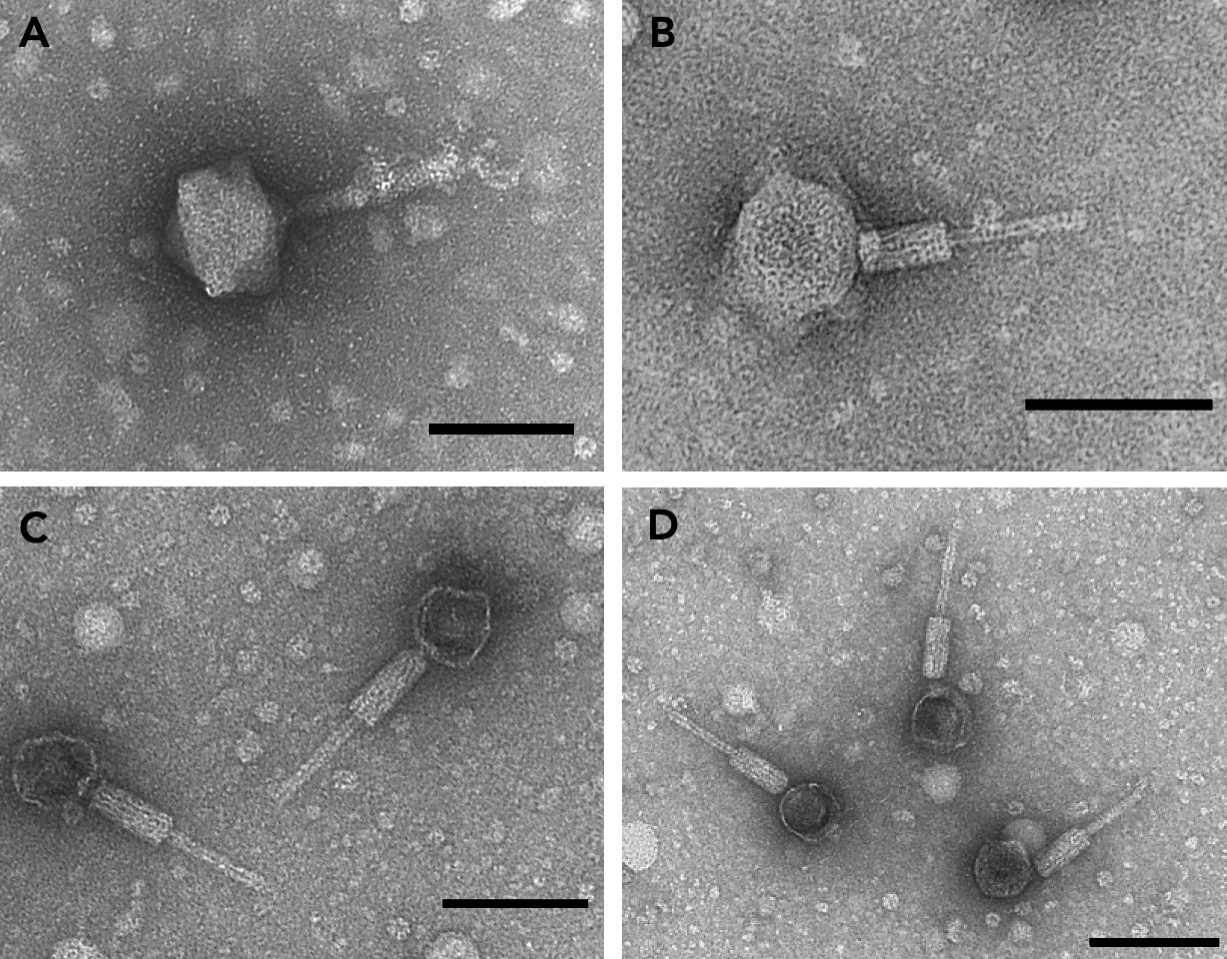
Transmission electron micrographs of phages Alfacinha1 (**A**), Alfacinha3 (**B**), Aval (**C**), and Acica (**D**). The scale bar represents 100 nm.

Soil samples from two commercial greenhouses growing lamb’s lettuce and one orchid greenhouse infested with *A. valerianellae* and *A. cattleyae*, respectively, were sampled to isolate bacteriophages. In total, four phages were isolated from the collected samples: Alfacinha1, Alfacinha3, both producing clear plaques, and Aval and Acica, producing turbid plaques. All phages show a myovirus morphology (contractile tails). While Alfacinha1 (Fig. 1A) and Alfacinha3 (Fig. 1B) have a head diameter of 101.9 + −2.1 nm (*N* = 5) and 67.5 + −3.3 nm (*N* = 24), respectively, Aval (Fig. 1C) and Acica (Fig. 1D) have a diameter of 55.1 + −3.5 nm (*N* = 15) and 54.6 + −2.4 nm (*N* = 13), respectively. Moreover, Alfacinha1 has convoluted tail fibers which are not observed for the other phages.

The host range of the phages was determined against a collection of bacterial strains as summarized by Table 1. Within this collection, different species of *Acidovorax* were included. As such, we could show that Alfacinha1 is a broad-spectrum phage, able to infect strains from two *Acidovorax* species, namely *A. valerianellae* and *A. cattleyae*. Interestingly, Acica, isolated from an orchid greenhouse, infects the same strains within the species of *A. cattleyae* as Alfacinha1. By contrast, Alfacinha3 and Aval are more specific and only infect *A. valerianellae* strains. While Alfacinha3 can infect seven strains, Aval has a very narrow host range and infects only three strains. Within the *A. cattleyae* species, two strains in collection show resistance to all isolated phages. In the case of *A. valerianellae*, 12 out of 19 strains (63%) are susceptible to the phages in collection.

**TABLE 1:** Host range of Alfacinha1, Alfacinha3, Aval, and Acica evaluated on different *Acidovorax* species and strains

Species	Strain	Alfacinha1	Alfacinha3	Aval	Acica
*A. anthurii*	CFBP 3232	**−**	**−**	**−**	**−**
*A. oryzae*	CFBP 2426	**−**	**−**	**−**	**−**
*A. citrulli*	LMG 5376	**−**	**−**	**−**	**−**
*A. cattleyae*	LMG 5286	**−**	**−**	**−**	**−**
	GBBC 705	**+**	**−**	**−**	**+**
	GBBC 1100	**−**	**−**	**−**	**−**
	GBBC 1148	**+**	**−**	**−**	**+**
	GBBC 1149	**+**	**−**	**−**	**+**
	GBBC 1303	**+**	**−**	**−**	**+**
*A. valerianellae*	CFBP 6945	**−**	**+**	**−**	**−**
	GBBC 3037	**+**	**−**	**−**	**−**
	GBBC 3038	**−**	**−**	**−**	**−**
	GBBC 3039	**+**	**−**	**−**	**−**
	GBBC 3042	**−**	**−**	**−**	**−**
	GBBC 3043	**−**	**−**	**+**	**−**
	GBBC 3129	**+**	**+**	**−**	**−**
	GBBC 3161	**−**	**+**	**−**	**−**
	GBBC 3208	**+**	**−**	**−**	**−**
	GBBC 3209	**−**	**+**	**−**	**−**
	GBBC 3340	**−**	**−**	**−**	**−**
	GBBC 3341	**−**	**−**	**+**	**−**
	GBBC 3342	**−**	**+**	**−**	**−**
	GBBC 3353	**+**	**+**	**−**	**−**
	GBBC 3354	**−**	**−**	**−**	**−**
	GBBC 3355	**−**	**−**	**−**	**−**
	GBBC 3356	**−**	**−**	**−**	**−**
	GBBC 3357	**+**	**+**	**+**	**−**
	GBBC 3358	**−**	**−**	**−**	**−**

### Taxonomical classification of the novel *Acidovorax* phages

**Fig 2:**
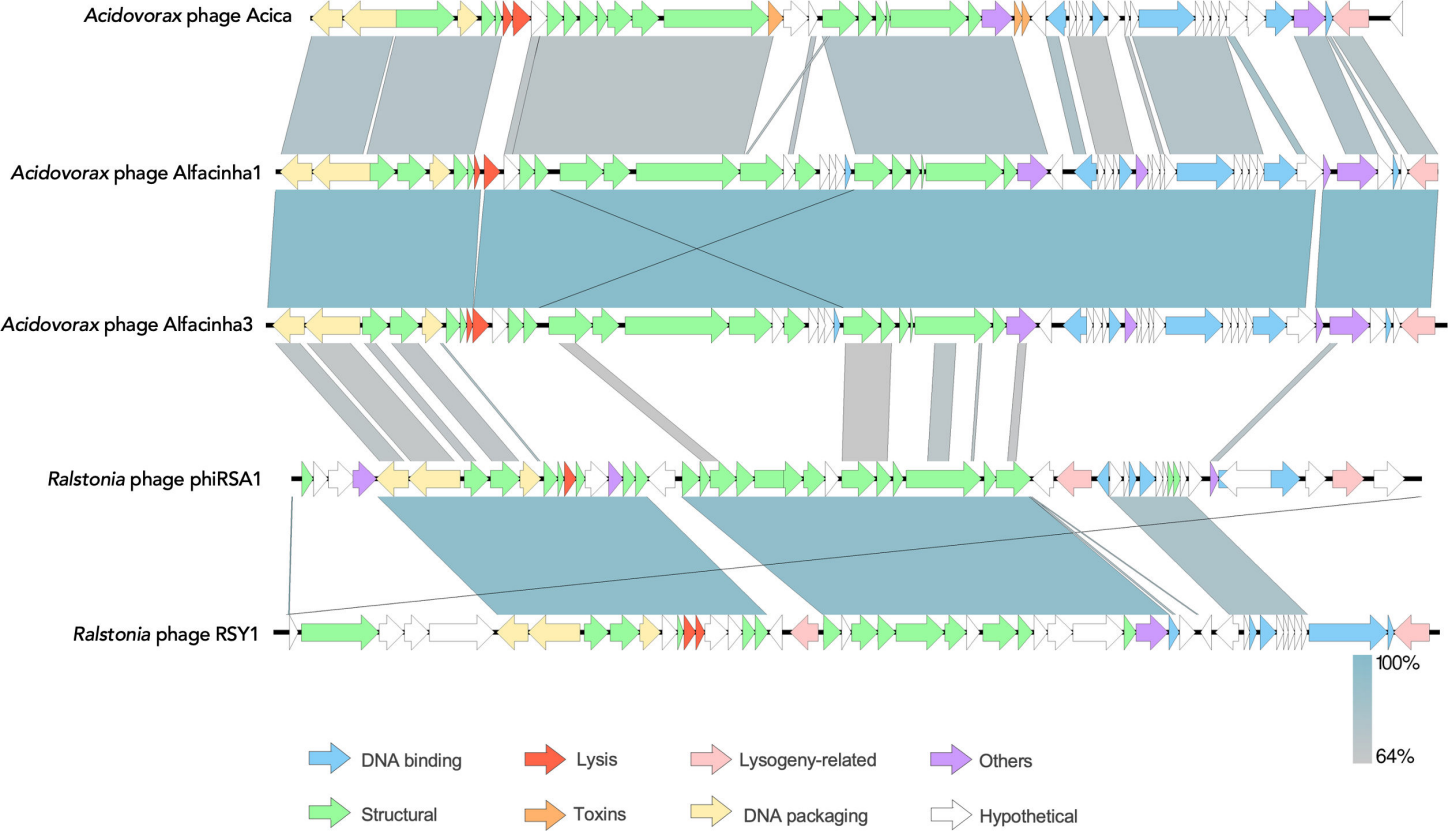
Genome map of Aval and of Acica, Alfacinha1, Alfacinha3, and the distantly related Ralstonia phages phiRSA1 and RSY1. The arrows indicate predicted ORFs and the direction of their translation: in white, encoding hypothetical proteins; blue, encoding DNA-associated proteins; green, encoding structural proteins, red, encoding lysis-related proteins; orange, encoding toxins; yellow, encoding DNA packaging proteins; pink, encoding lysogeny related proteins; and purple, encoding other proteins. Generated with EasyFig.

The four phages in collection were whole-genome sequenced. A Viptree analysis shows that Aval is an orphan phage with no homology to other described viruses in public databases (Fig. S1). A VIRIDIC analysis of the closest relatives showed that the phage indeed does not share homology and should therefore be considered a new species within a new genus. As shown in the heatmap presented in Fig. S2, three clusters can be distinguished. One comprises *Streptomyces* phages SF1, SF3, and VWB, another with the *Propionibacterium* phages PFR1 and PFR2 (*Pulverervirus* genus members), and the third one with members of the *Dolichocephalovirinae* subfamily, including *Caulobacter* phages CCrColossus, CCrRogue, CCrSwift, phiCbK, CCrKarma, and CCrMagneto. Interestingly, Aval does not belong to any cluster, thus representing a potential orphan phage genus. This phylogenetic clustering is further supported by a neighbor-joining tree based on the major capsid protein from these phages. Based on this taxonomical analysis, we can hypothesize that *Acidovorax* phage Aval is a temperate phage (phage.AI). Indeed, zooming in on the genome map of the phage and the individual open reading frames, several ORFs encoding lysogeny-associated genes are annotated (Fig. 2). More specifically, Aval encodes an integrase, commonly found in temperate phages integrating into their host’s genome using site-specific recombination. Also, CI and CII repressors are encoded, further supporting the temperate lifestyle of Aval. An analysis of the large terminase demonstrated that Aval most likely uses a cos-like packaging strategy (Fig. S3). The phage also carries a gene encoding a pyocin, a bacteriocin potentially contributing to lysogenic conversion aiding the host in interspecies competition, further excluding its potential for phage biocontrol purposes.

By contrast, a Viptree analysis of Alfacinha1, Alfacinha3, and Acica shows that these phages are members of the *Peduoviridae* family and constitute two new phage genera and two new species within this family (Fig. S4). A VIRIDIC analysis of this family demonstrates that indeed Acica shares less than 70% sequence identity with Alfacinha1 and Alfacinha3, suggesting it represents yet another phage genus (Fig. S5, packaging strategy Fig. S6). Interestingly, the *Acidovorax* phages share to some extent sequence identity with the *Tigrvirus* and *Citexvirus* genera, suggesting a distant relatedness to these genera.

The genome maps of Alfacinha1, Alfacinha3, and Acica illustrate the relatedness of these phages (Fig. 2). Interestingly, one of the most striking differences between the three phages is the absence of the BrnT/BrnA toxin-antitoxin system (gp27 and gp28), a ribonuclease toxin system, encoded by Acica and not by Alfacinha1 or Alfacinha3. However, all phages encode a site-specific integrase (arm-like integrase), potentially excluding them as a biocontrol agent. Despite the presence of this integrase in the genomes of Alfacinha1 and Alfacinha3, we decided to further evaluate the potential of this phage species as a biocontrol agent based on its host range as we did not have an indication of a temperate lifestyle during the microbiological characterization of the phage. Indeed, we were not able to isolate lysogenic bacteria. Furthermore, other members of the *Peduoviridae* have been demonstrated to be useful as biocontrol agents in different settings (42, 43).

### Microbiological characterization of Alfacinha3 as a potential agent for Biocontrol applications

**Fig 3:**
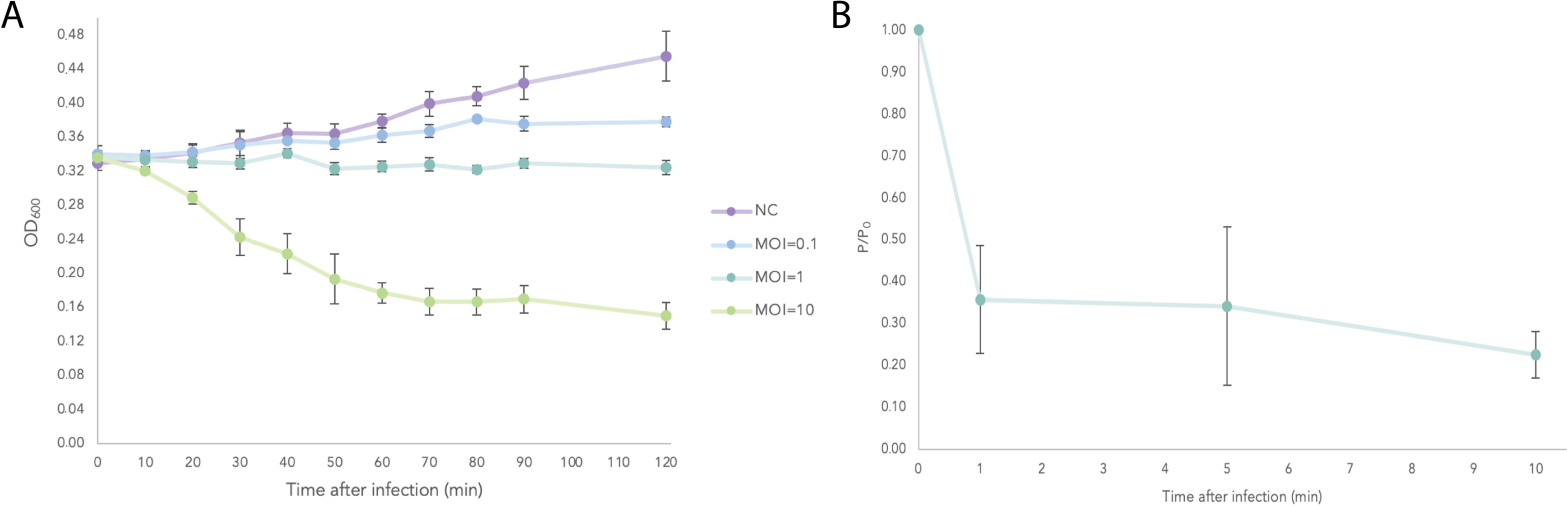
Adsorption and infection curves of Acidovorax phage Alfacinha3. (**A**) Infection curves of *A. valerianellae* GBBC 3161 infected with phage Alfacinha3 at different MOIs. The variation in optical density (OD_600_) during growth of *A.valerianellae* GBBC 3161 with different concentrations of phage is followed through time. The negative control is indicated with lilac, MOI 0.1 with blue, MOI 1 with turquoise, and MOI 10 with light green. Error bars indicate the SD and are based on three independent repeats. (**B**) Adsorption curve of Alfacinha3 to the host strain *A.valerianellae* GBBC 3161, with MOI = 0.01. The ratio of non-adsorbed phages (**P**) to the initial titer (**P0**) is followed through time. Error bars indicate the SD and are based on three independent repeats.

Based on the molecular microbiology selection criteria, Alfacinha1 and Alfacinha3 were chosen as potentially suitable biocontrol agents. We therefore evaluated the adsorption kinetics and the ability of this phage species to lyse a bacterial culture in liquid medium, as illustrated in Fig. 3. Our assay showed that after 1 minute, about 35% of the initial Alfacinha3 population remained non-adsorbed, indicating that 65% of phages were successfully adsorbed to the bacterial cells in liquid culture. Ten minutes after adding the phage, over 70% of the phage population was adsorbed as the percentage of free phage dropped to 30%. Given these parameters, we could calculate that the adsorption constant for Alfacinha3 while infecting GBBC3161 was 3.52 × 10^−9^ mL/min after 1 minute. Alfacinha3 also lyses growing bacterial cultures when added in a ten-to-one ratio of phage to bacteria (MOI 10). After 20 minutes, the optical density dropped significantly compared to the negative control (growth of GBBC3161 without phage). However, at lower concentrations of Alfacinha3 (MOI of 0.1 and 1), no lysis event could be observed, but rather a reduction of the bacterial growth compared to the negative control.

### Alfacinha3 lowers bacterial titers on seeds and improves seedling germination

**Fig 4:**
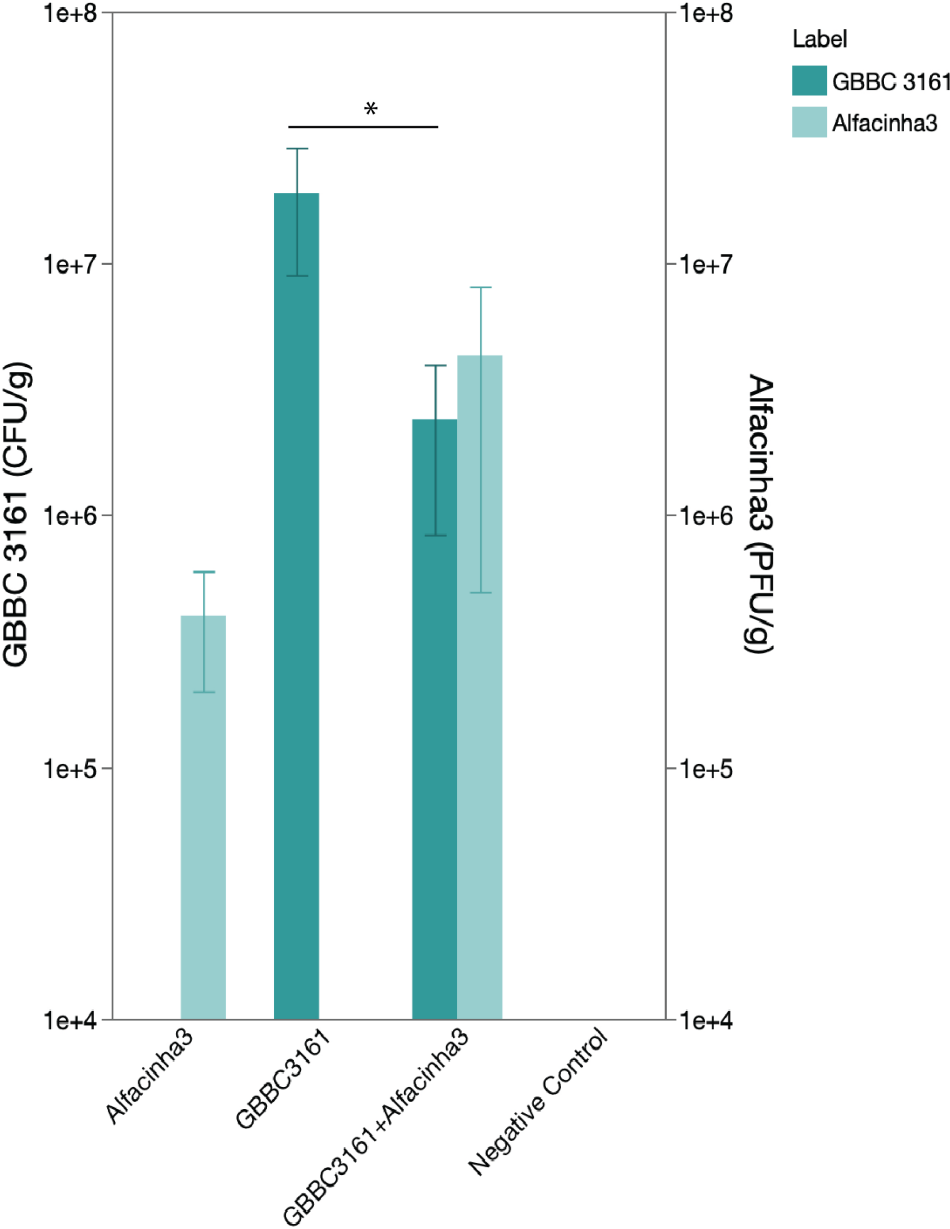
Bacterial (**A**) and phage (**B**) concentration on the seeds for the four different conditions—negative control, bacteria only (infected with *A. valerianellae* strain GBBC 3161 at 10^8^ CFU/mL), phage only (primed with Alfacinha3 at 10^9^ PFU/mL), and bacteria plus phage (GBBC 3161 + Alfacinha3). Error bars indicate the SD and are based on three independent repeats. Statistical support is based on *t*-test, showing significant difference in (**A**) (*P* < 0.05) and no significant difference in (**B**) (*P* = 0.15).

**Fig 5:**
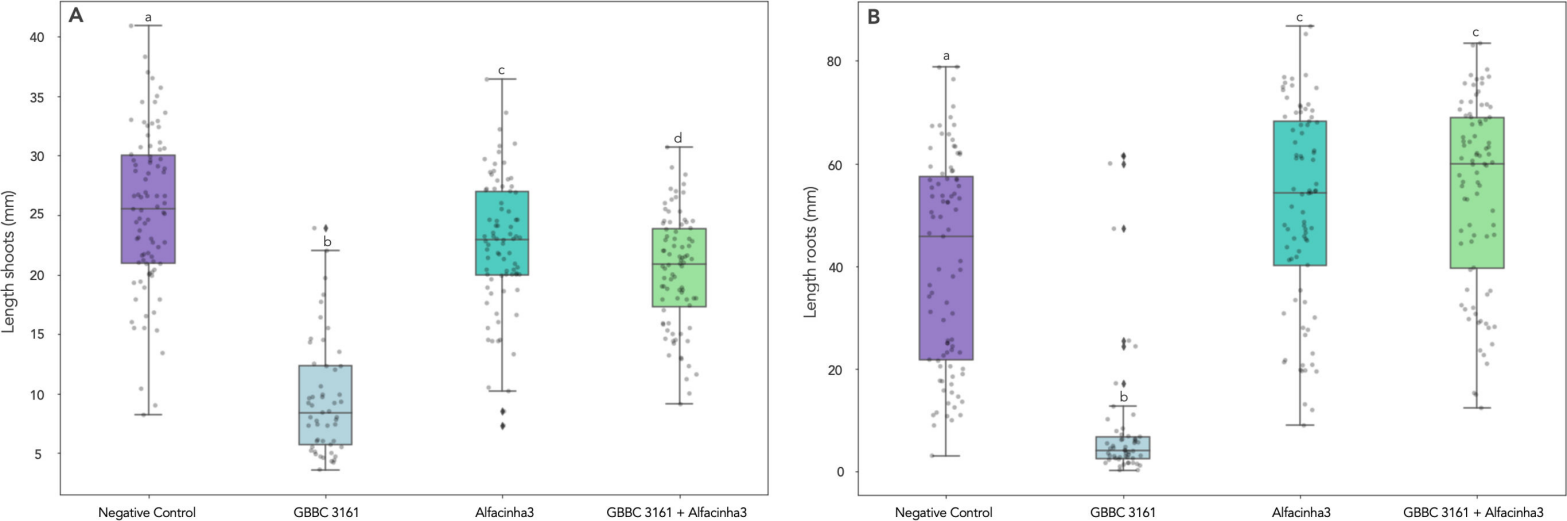
Measurements of the shoot (**A**) and root (**B**) length after germination in four different conditions: negative control; bacteria only (infected with *A. valerianellae* strain GBBC 3161 at 10^8^ CFU/mL); phage only (primed with Alfacinha3 at 10^9^ PFU/mL); and bacteria plus phage (GBBC 3161 + Alfacinha3). The results are based on three independent repeats, each one using 30 seeds per condition. Statistical relevance is represented by the connecting letters based on the nonparametric comparisons performed with Wilcoxon method (*P* = 0.05).

As *A. valerianellae* is seed-borne, the potential of Alfacinha3 was evaluated during a seed steeping assay. Lamb’s lettuce seeds were soaked in a bacterial suspension, dried, and incubated in a watery solution for 24 hours in either the presence or absence of phage. We determined the bacterial load as the number of colony-forming units per gram of seed as well as the number of plaque-forming units per gram of seed. After incubation, we observed a significant reduction in the bacterial concentration on the seeds (Fig. 4). Interestingly, the phage concentration significantly increased as well, indicating successful propagation of the phages during steeping (Fig. 4). We further assessed the effects of the phage treatment on the development of lamb’s lettuce seedlings (Table 2). In total over the three biological replicates, 93.3% of seeds germinated after phage treatment compared to 58.9% without phage treatment. Seedlings germinating from seeds infested with *A. valerianellae* were significantly smaller (both the shoots and the roots) compared to the negative control (Fig. 5). Seedlings that received a phage steeping earlier showed both improved shoot and root length. However, a small but measurable decrease in the shoot growth of the seedlings was observed compared to the control, suggesting a minor influence of the phage treatment. Interestingly, the root length increased in the presence of phage. Plants that received phage in the absence of bacteria showed a significant increase in lateral roots (Fig. S7). We calculated the vigor index as a measure to assess shoot length, root length, and the percentage of seeds that germinate successfully. There was a drastic increase of the vigor index when seeds were steeped with phage (62.1 for non-treated seeds vs 1054.3 for phage-treated seeds). These results suggest that phages are indeed applicable in this system to reduce the impact of epiphytically infested lamb’s lettuce seeds.

**TABLE 2:** Measurements of seedlings grown for 22 days from each condition—negative control, bacteria only (strain GBBC 3161), phage only (Alfacinha3), and bacteria plus phage (GBBC 3161 + Alfacinha3)^*a*^

	Mean shoot length (mm)	Mean root length (mm)	Germination rate (%)	Vigor index^*a*^
Negative control	25.4 ± 4.34	40.9 ± 4.93	96.7 ± 5.77	1044.1 ± 78.12 ^A^
GBBC 3161	9.7 ± 2.99	8.5 ± 7.78	58.9 ± 3.85	62.1 ± 66.53 ^B^
Alfacinha3	22.5 ± 3.40	50.7 ± 6.60	90.0 ±12.02	1085.6 ± 358.58 ^A^
GBBC 3161 + Alfacinha3	20.1 ± 3.02	54.2 ± 6.97	93.3 ± 6.67	1054.3 ± 274.67 ^A^

^*a*^ The vigor index was calculated as the product of the shoot and root lengths with the percentage of seed germination. The results are based on three independent repeats, each one using 30 seeds per condition. Statistical support is given for the vigor index parameter by a connecting letters report (A and B; different letters indicate statistically different groups), based on the comparisons performed with Wilcoxon method (*P* = 0.05).

### Lipopolysaccharides in phage suspensions influence significantly seedling development

**Fig 6:**
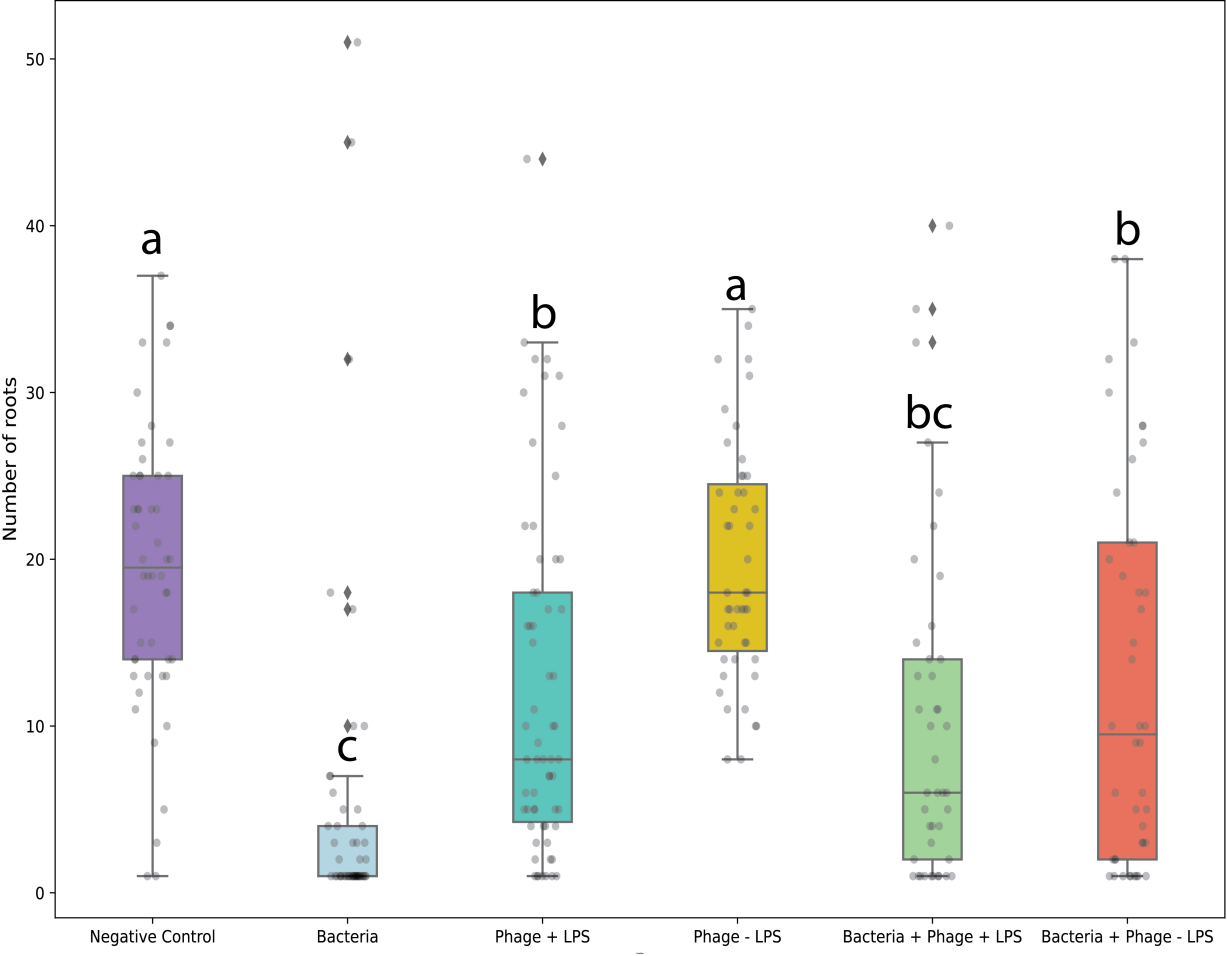
Number of roots per seedling for each condition: negative control, bacteria (*A. valerianellae* GBBC 3357 at 10^8^ CFU/mL), Phage + LPS (Alfacinha1 with LPS at 10^9^ PFU/mL), Phage-LPS (Alfacinha1 cleared from LPS), Bacteria + Phage + LPS (*A. valerianellae* GBBC 3357 with Alfacinha1 and LPS in the phage solution), and Bacteria + Phage LPS (*A. valerianellae* with Alfacinha1 cleared from LPS). The results are based on four independent repeats with 12 seedlings per replicate. Connecting letters were determined by a Tukey-Kramer HSD test with corrections for multiple comparisons (*P*-value < 0.05).

We further evaluated the effects of residual endotoxins such as LPS in phage suspensions on the development of lamb’s lettuce seedlings. To this end, we cleared LPS from an Alfacinha1 phage solution by means of an EndoTrap purification column. First, seeds were inoculated with *A. valerianellae*, followed by a phage treatment. For the phage treatment, both the concentrated lysate and LPS-cleared phage preparation were used. Next, the seeds were germinated and grown. After 4 weeks, we counted the number of lateral roots per seedling (Fig. S8) for all conditions. Here, we observed a significant effect of phage suspensions treated with crude phage lysates, and phage solutions cleared from LPS on the number of lateral roots per seedling (Tukey-Kramer HSD, *P*-value < 0.05, Fig. 6, connecting letters report). There was no significant difference for the number of lateral roots counted between the negative control and seeds treated with an Alfacinha1 solution cleared from LPS. When LPS was not removed from the phage suspension, there was a significant difference with the negative control, suggesting that residual LPS impacts root development in lamb’s lettuce. Inoculating seeds with *A. valerianellae* drastically reduced the number of roots. Treating the seeds with Alfacinha1 cleared from LPS significantly improved root development. Yet, there was no significant difference observed in the number of roots between the objects inoculated with *A. valerianellae* with and without Alfacinha1 containing residual LPS. In summary, this assay demonstrated that residual LPS in phage solutions impacts the root development of lamb’s lettuce.

## DISCUSSION

Members of the *Acidovorax* genus infect a wide diversity of crops and ornamentals. Despite the importance of these pathogens, limited disease management strategies are in place to control disease outbreaks. Biological control with natural antagonistic micro-organisms inhabiting the rhizosphere has been exploited against different plant diseases including the use of bacteria (44), yeasts (45), and bacteriophages (22, 46).

Here, we evaluated the potential of bacteriophages to control disease in lamb’s lettuce. We characterized four phages isolated from diseased greenhouses. Our current phage collection consists of one orphan phage Aval and two new phage genera part of the *Peduoviridae* family (Acica, Alfacinha1, and Alfacinha3). Remarkable is the difference in host range between Alfacinha1 and Alfacinha3, both part of the same species. However, we observed a difference in the phages’ lysis cassette, suggesting that alterations in the phage endolysins could contribute to differences in the phage host range. Indeed, endolysins have been reported to contribute to host specificity and are subjected to evolutionary dynamics (47, 48).

Seed treatment was proposed as a control strategy for *A. valerianellae* since transmission by contaminated seeds is one major infection source. The use of bacteriophages could provide several advantages when compared with the available seed treatments, as phages are able to remain infective for long periods around the seeds, even after germination, and represent a more selective and ecologically sustainable strategy. The use of phage-coated seeds has been previously reported to be an effective approach in the biocontrol of another *Acidovorax* species, *A. citrulli*, being able to reduce the development of BFB. Indeed, treatment showed to increase the germination rate of watermelon-infested seeds from 55% to 88% and the plant survival rate after 3 weeks from 15% to 100% (23). As such, in this study, a seed bioassay was performed to test the efficacy of the Alfacinha1 and Alfacinha3 phage species in the control of *A. valerianellae* on seeds and demonstrated to be applicable in this system. A cocktail of both Alfacinha phages was thus far not assessed or the emergence of phage-insensitive mutants but could be a valid option in future experiments since both phages, although belonging to the same species, might behave differently against the target bacteria and might work synergistically.

Interestingly, plants from phage-primed seeds showed significant differences in morphology. Despite shorter shoots they presented considerably longer and more developed roots, with increased lateral root density (as verified in Figure 6). This indicates that the interaction with the phage solution potentially induces a plant response. Plants are known to respond to a variety of stimuli, including pathogen-associated molecular patterns, such as lipopolysaccharides, peptidoglycans, and bacterial flagellin, which trigger a defense response to protect the plant from invading pathogens (49). The detection of quorum sensing (QS) signaling molecules of microorganisms has also been reported to induce a plant response. For instance, a recent study showed that diketopiperazines (QS signal molecules) promote lateral root development and root hair formation in *Arabidopsis thaliana* by enhancing the polar transport of the plant hormone auxin from the shoots to the roots. This led to the accumulation of auxin at the root tip that, in turn, accelerated root growth (50). Therefore, we may hypothesize that the phage stock used in the seed coating still contains bacterial compounds that cause a plant response. The bacterial compounds could include endotoxins, such as LPS, that could not be completely removed by polyethylene glycol (PEG) precipitation (51), or QS signaling molecules, that induce hormone signaling, promoting the specific growth and development of the root. To test this hypothesis, we cleared an Alfacinha1 suspension from LPS and evaluated its effect on root development. Our assay demonstrated that indeed residual LPS negatively impacts root development in lamb’s lettuce. This observation makes us critically aware of the impact of residual LPS in phage applications and the need for additional purification steps in designing phage applications. Alternative methods to PEG precipitation, including combinations of dead-end filtration, cross-flow filtration, and affinity chromatography have also been proposed (51).

As demonstrated in our seed assay, the application of phages suggests a growth promotion of lamb’s lettuce seedlings, indicating that plants potentially respond to the presence of phage. However, this correlation should still be confirmed for example by investigation of plant hormone and key regulatory gene expression levels in inoculated plants with and without phage treatment in future experiments. This relationship has already further been demonstrated in brassicas. During metabolic profiling of plants treated with phage Xccφ1, the authors observed differences between the phage-treated plants and the water control. More specifically, plants contained higher levels of citrate, and lower concentrations of valine, leucine, isoleucine, threonine, lysine, alanine, GABA, and pipecolate (52). These results are, to the best of our knowledge, the first indication that plants indeed respond to the presence of phage. Our results provide further evidence for the existence of this interaction. Future research should further investigate this interaction and especially the role of the plant’s immune system in this respect.

## Data Availability

Phage genomes are available at NBCI under the accession numbers OP131587, OP131588, OP131589, and OP131590.

## SUPPLEMENTAL MATERIAL

Supplemental figures spectrum.00993-24-s0001.docx Fig. S1 to S8.

ASM does not own the copyrights to Supplemental Material that may be linked to, or accessed through, an article. The authors have granted ASM a non-exclusive, world-wide license to publish the Supplemental Material files. Please contact the corresponding author directly for reuse.
