# Supplementary material for: Characterization of four Acidovorax phages and their potential in phage biocontrol for lamb’s lettuce seed decontamination
Source: Microbiol Spectr. 2024 Oct 28;12(12):e00993-24. doi: 10.1128/spectrum.00993-24 (PMC11619371; doi:10.1128/spectrum.00993-24)
Supplement: Supplemental figures — Fig. S1 to S8. [file spectrum.00993-24-s0001.docx]

***Supplementary information***


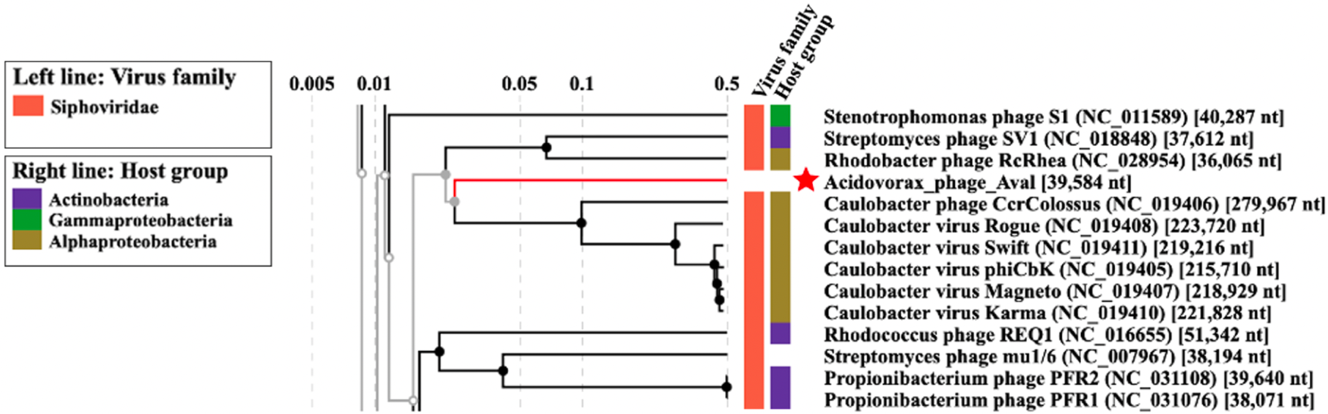


**Supplementary Figure 1 Proteomic tree based on global genomic similarity relationships between Aval and other known phages, predicting the virus family and host group.** Only the section with the most related phages of the tree is presented in this figure. Aval is indicated with a red star. Generated in ViPTree.


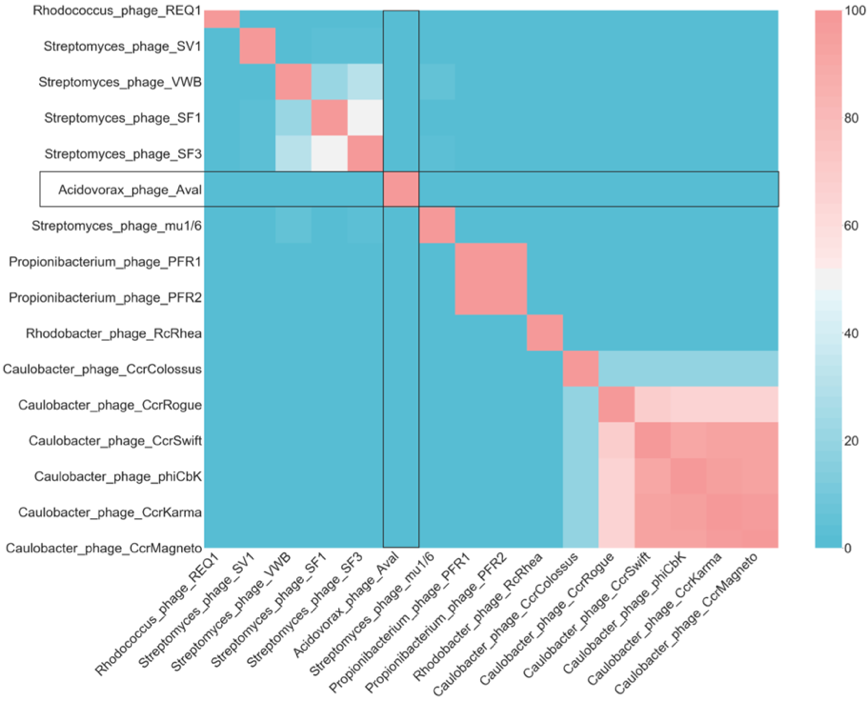


**Supplementary Figure 2 Heatmap based on the calculated sequence similarities (using VIRIDIC) between Aval and its most closely related phages.**


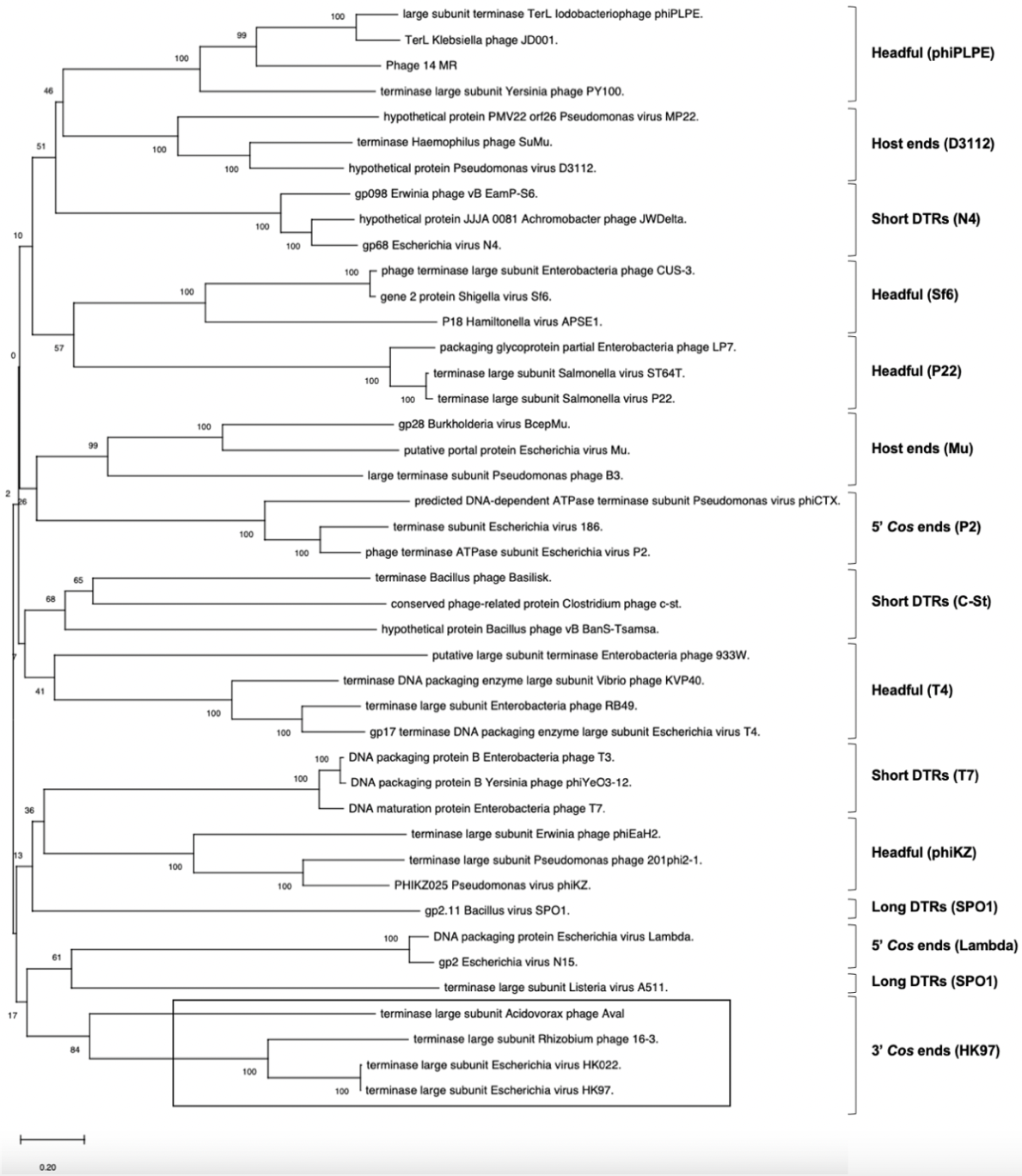


**Supplementary Figure 3 Neighbor-joining tree of large terminase protein sequences of Aval and phages with a known packaging.** The black rectangle indicates Aval’s cluster. The brackets indicate the packaging strategies experimentally determined for each cluster, according to Merrill et al.^53^. Bootstrap values are for 1000 trials. The scale bar shows 0.2 amino acid substitutions per site. Generated in MegaX.

^52^
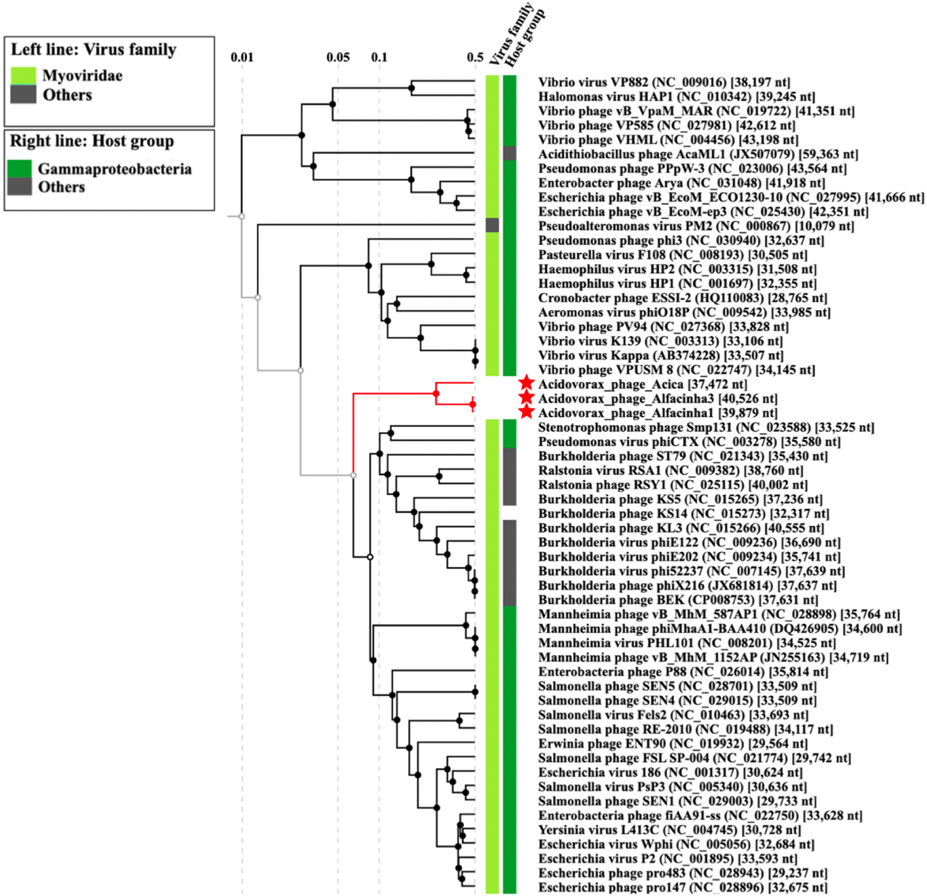


**Supplementary Figure 4 Proteomic tree based on global genomic similarity relationships between Acica, Alfacinha1, Alfacinha3 and other known phages, predicting the virus family and host group.** Only the section with the most related phages of the tree is presented in this figure. Alfacinha1, Alfacinha3 and Acica are indicated with red stars. Generated in ViPTree.


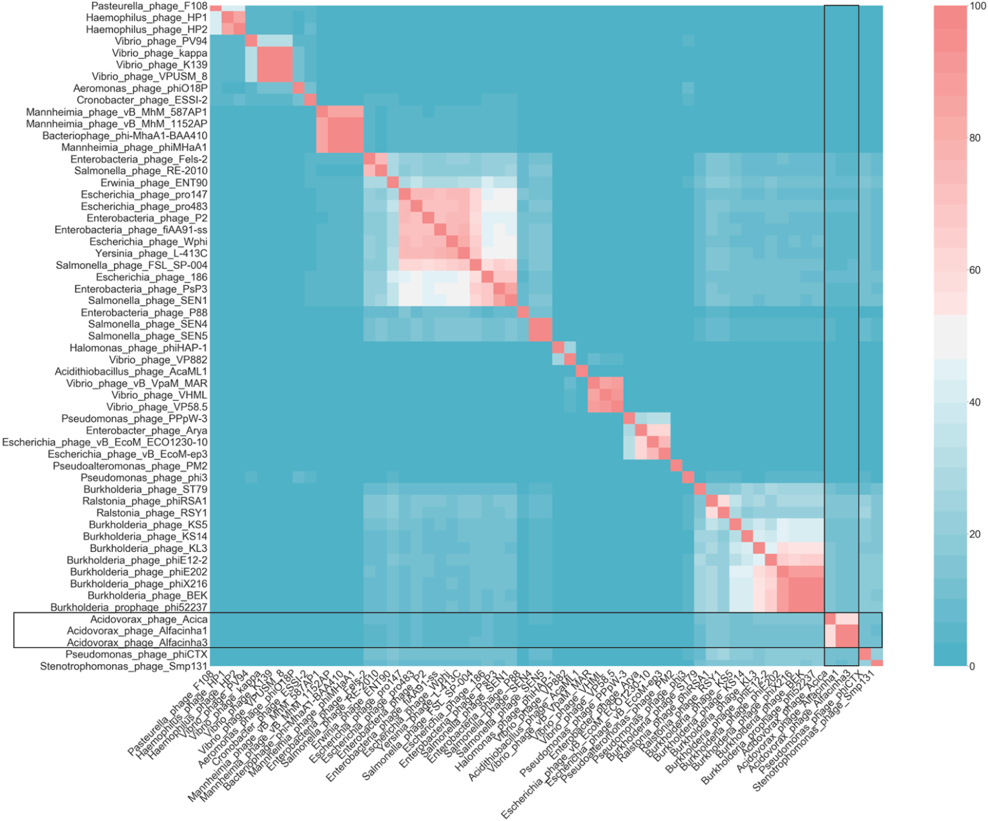


**Supplementary Figure 5 Heatmap based on the calculated sequence similarities (using VIRIDIC) between Alfacinha1, Alfacinha3, Acica and their most closely related phages.**


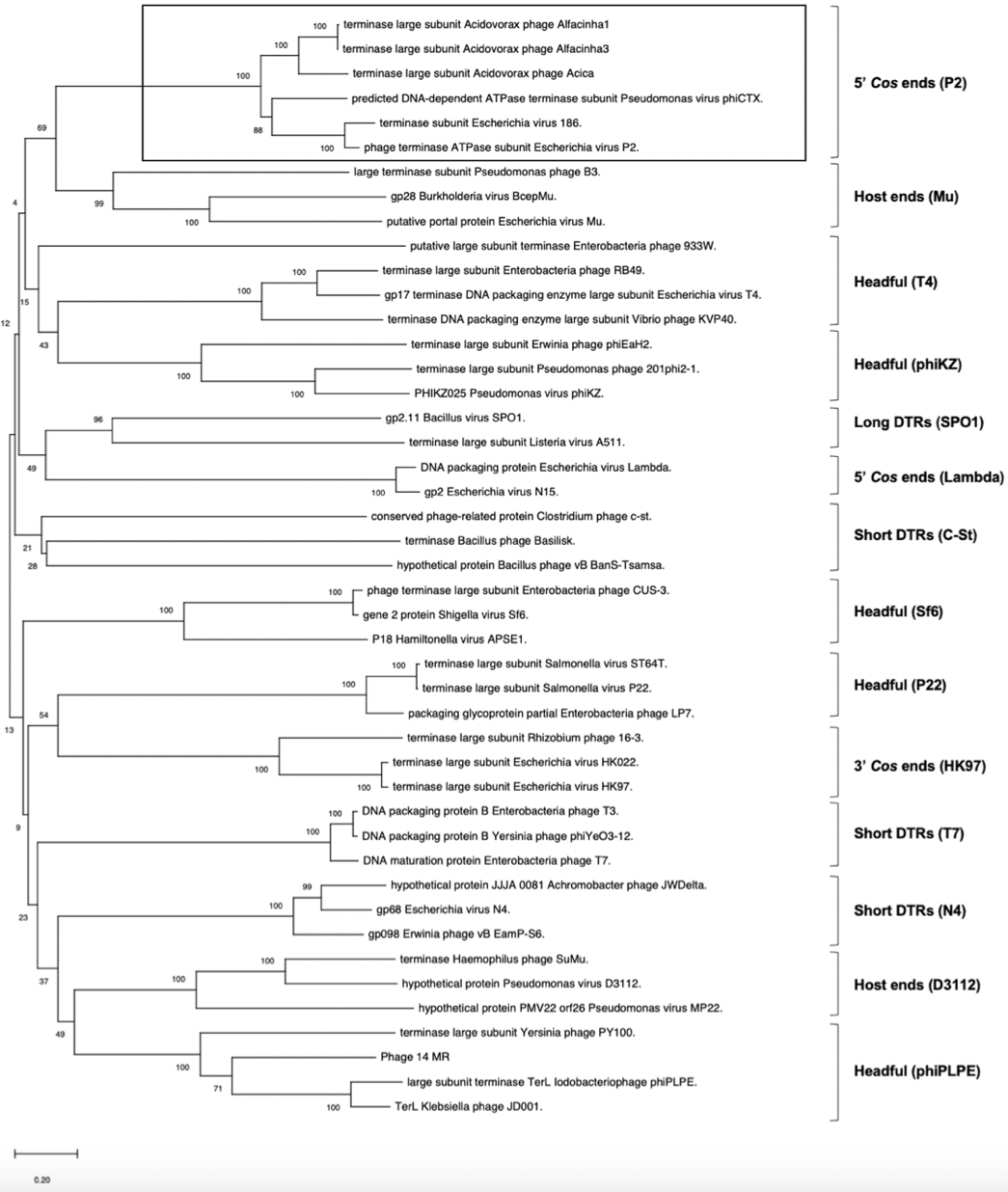


**Supplementary Figure 6 Neighbor-joining tree of large terminase protein sequences of Alfacinha1, Alfacinha3, Acica and phages with known packaging strategy.** The black rectangle indicates the Alfacinha and Acica cluster. The brackets indicate the packaging strategies experimentally determined for each cluster, according to Merrill et al. ^53^. Bootstrap values are for 1000 trials. The scale bar shows 0.2 amino acid substitutions per site. Generated in MegaX.


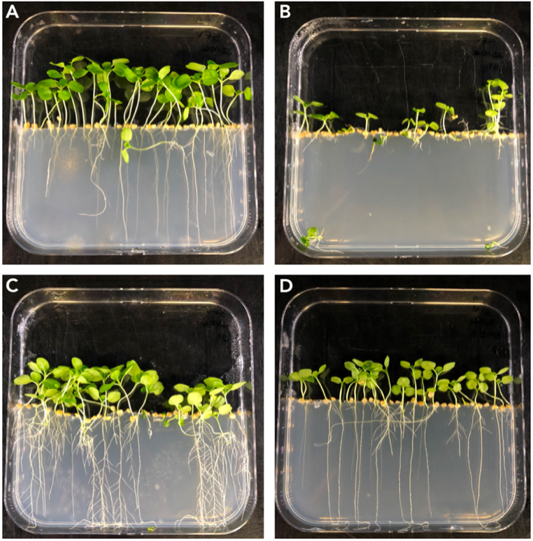


**Supplementary Figure 7 Lamb’s lettuce seedlings after germination under four different conditions: A) negative control; B) bacteria only (infected with A.valerianellae strain GBBC 3161 at 10^8^ CFU/mL); C) phage only (primed with Alfacinha3 at 10^9^ PFU/mL) and D) bacteria plus phage (GBBC 3161 + Alfacinha3).**

**
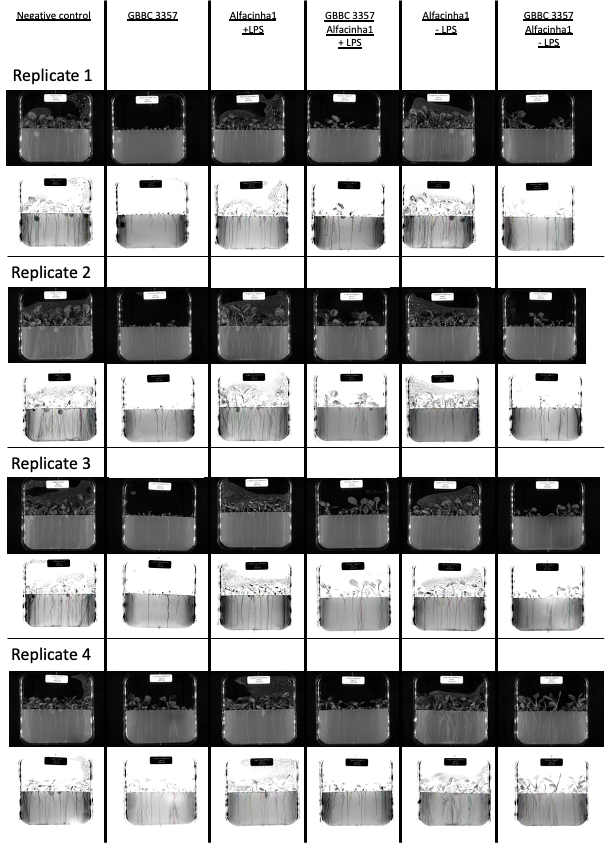
**

**Supplementary Figure 8 Lamb’s lettuce seedlings after germination under six different conditions and four replicates: (from left to right) Negative control, GBBC 3357, Alfacinha1 + LPS, GBBC 3357 and Alfacinha1 + LPS, Alfacinha1 – LPS, and GBBC 3357 and Alfacinha1 – LPS.** On top is the unmodified picture, on the bottom is the inverted picture with enhanced contrast and annotated roots (as obtained with Fiji).
